# Aroxybutynin and atomoxetine (AD109) for the treatment of obstructive sleep apnea: Rationale, design and baseline characteristics of the phase 3 clinical trials

**DOI:** 10.1016/j.conctc.2025.101538

**Published:** 2025-08-17

**Authors:** Luigi Taranto-Montemurro, Sanjay R. Patel, Patrick J. Strollo Jr., John Cronin, John Yee, Huy Pho, Andrea Werner, Ron Farkas

**Affiliations:** a Apnimed, Inc., Cambridge, MA, USA; b Division of Pulmonary, Allergy, Critical Care, and Sleep Medicine, University of Pittsburgh, PA, USA

## Abstract

**Introduction:**

Two key factors leading to obstructive sleep apnea (OSA) pathogenesis include relaxation of upper airway muscles at sleep onset and their insufficient reactivation during obstructive events. Medications that address this neuromuscular dysfunction by increasing upper airway tone during sleep represent a potential strategy for mitigating OSA.

**Methods:**

AD109 is an investigational, once-daily oral agent taken at bedtime that combines an antimuscarinic, aroxybutynin (2.5 mg), with a selective norepinephrine reuptake inhibitor, atomoxetine (75 mg). LunAIRo (NCT05811247) and SynAIRgy (NCT05813275) are two ongoing, placebo-controlled 51-week and 26-week phase 3 clinical trials, respectively, investigating the efficacy and safety of AD109 to treat mild to severe OSA. Participants include adults with an apnea-hypopnea index with 4% desaturation (AHI_4_) >5 who either refuse or fail to tolerate positive airway pressure. Participants (LunAIRo: N = 660; SynAIRgy: N = 646) were randomized 1:1 to receive AD109 or placebo. We hypothesize that AD109 will significantly reduce AHI_4_ and symptomatic fatigue compared to placebo in people with OSA. The primary outcome for both trials is the change from baseline to Week 26 in AHI_4_ in the AD109 arm versus placebo. Key secondary outcomes include changes from baseline in oxygen desaturation index with 3% desaturation, hypoxic burden based on 4% desaturation, Patient Reported Outcome Measurement Information System (PROMIS)-Fatigue, and proportion of participants with ≥50% reduction in AHI_4_ at Week 26.

**Discussion:**

LunAIRo and SynAIRgy are fully enrolled, large Phase 3 clinical trials designed to confirm and extend our understanding of the safety and efficacy of AD109, a combination oral drug targeting the underlying neuromuscular dysfunction contributing to upper airway muscle collapse during sleep in adults with OSA.

## 1 Introduction

Obstructive sleep apnea (OSA) is a common, serious, chronic disease affecting nearly a billion individuals worldwide, impacting quality of life and linked to significant long-term comorbidities and early mortality [1,2]. OSA is characterized by sleep-related neuromuscular dysfunction and predisposing anatomic abnormalities: decreased activity of pharyngeal dilator muscles during sleep in the context of a narrowed pharyngeal airway. Few treatment options are available for OSA, with patients most commonly treated with continuous positive airway pressure (CPAP) [3]. Although CPAP is usually efficacious because it mechanically splints the airway open, limited adherence remains a major problem due to many factors including discomfort, interference with sleeping position, noise, claustrophobia, inconvenience, and perceived stigma [4]. A number of drugs including modafinil, armodafinil, solriamfetol, and pitolisant are approved for the narrow indication of residual excessive daytime sleepiness (EDS) despite maximal effort to treat with CPAP [5]. These drugs do not address the underlying neuromuscular pathophysiology of OSA (i.e., upper airway collapse and obstruction), and only deal with one of the consequences, EDS, in a relatively small subgroup of OSA patients.

Increasing the activity of upper airway dilator muscles, such as the genioglossus, and thereby reducing sleep-related airway muscle hypotonia, is considered a promising strategy to treat OSA [6,7]. Preclinical work suggests that the main cause of sleep-related hypotonia of the pharyngeal muscles is the progressively reduced firing frequency of norepinephrine neurons, which innervate these pharyngeal dilator muscles, from wakefulness to non-rapid eye movement (NREM) sleep, and from NREM to rapid-eye-movement (REM) sleep stages [[8], [9], [10]]. While noradrenergic withdrawal is thought to be the main cause of pharyngeal hypotonia in NREM sleep [8], there appears to be additional muscarinic inhibitory mechanisms that cause further reduction in dilator muscles activity especially during REM sleep [11].

AD109 is a combination of the antimuscarinic, aroxybutynin, (R-enantiomer of oxybutynin), and the selective norepinephrine reuptake inhibitor, atomoxetine. Rosenberg et al. tested for the first time two doses of AD109 (2.5/37.5 mg and 2.5/75 mg of aroxybutynin/atomoxetine) in a crossover trial of participants with mild-to-moderate OSA (apnea-hypopnea index [AHI_4_] between 5 and 20 events/h). The combination showed a dose-dependent and statistically significant reduction in OSA severity compared to placebo after acute (1-night) administration. Recently, the MARIPOSA Phase 2 randomized, placebo-controlled parallel-arm clinical trial assessed the efficacy of AD109 (2.5/75 mg) over one month of treatment in 211 participants having a baseline AHI_4_ between 10 and 45 events/h. The study demonstrated that AD109 reduced AHI_4_ by a mean of 47.1% compared to placebo, with >70% decrease in about a quarter of participants and a decrease >90% in about 10% of participants [12]. In addition to reducing AHI_4_, AD109 demonstrated a statistically significant improvement in fatigue compared to placebo, assessed using the Patient Reported Outcome Measurement Information System (PROMIS) scale [12].

The LunAIRo and SynAIRgy phase 3 clinical trials have been designed to confirm and extend the objective and subjective efficacy of AD109 in OSA with longer-term use. These trials will provide a comprehensive evaluation of AD109's therapeutic benefits within the context of two rigorously conducted phase 3 trials.

## 2 Methods

### 2.1 Study overview

**Fig. 1:**
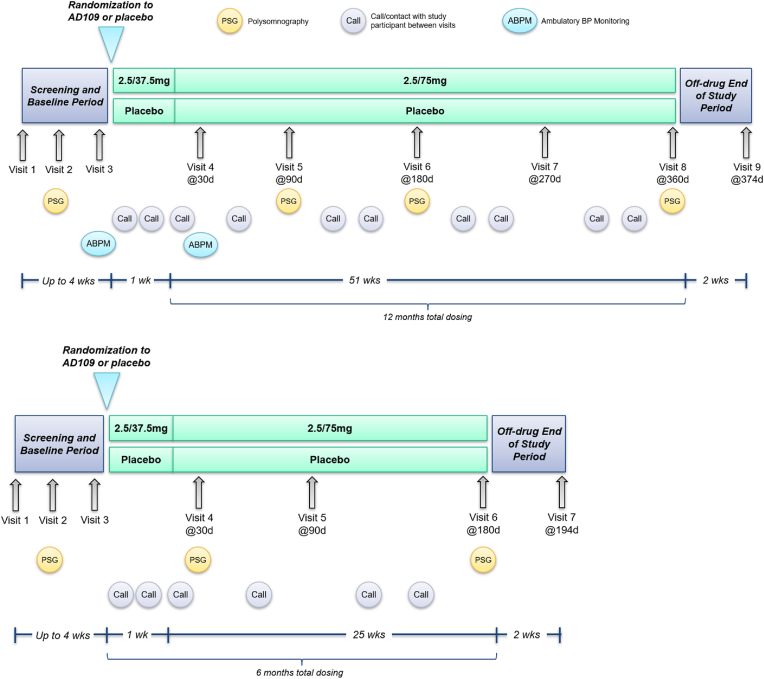
Diagrams of the LunAIRo (top) and SynAIRgy (bottom) studies.

LunAIRo (NCT05811247) and SynAIRgy (NCT05813275) are two independent, multicenter, randomized, double-blinded, placebo-controlled parallel arm clinical trials comparing the efficacy and safety of AD109 versus placebo in adults with OSA who are intolerant to or currently refuse PAP. The study schema of the LunAIRo and SynAIRgy studies are shown in Fig. 1. Objective efficacy data are gathered with in-laboratory polysomnograms (PSG) performed at 13, 26, and 51 weeks for the LunAIRo study, and at 4 and 26 weeks for the SynAIRgy study. Patient reported outcomes (PROs) are assessed at the same intervals as the PSGs. Safety data are assessed throughout the 51-week duration of the LunAIRo study and the 26-week duration of the SynAIRgy study. Participants completing either of these studies are eligible for an optional open label extension (OLE) study of AD109 in which safety is assessed for up to an additional year (**Supplement Information**).

For both trials, a screening visit and PSG is conducted to establish that each participant meets study enrollment criteria. Eligible participants are randomized to one of two parallel treatment arms. Randomization is stratified according to baseline AHI_4_ (<15, 15 to 30, >30) and PROMIS Short Form v1.0-Fatigue 8a raw score (PROMIS-Fatigue; <25 versus ≥25; a raw score of 25 is equivalent to a T-score of 58.5); to ensure a representative distribution of baseline AHI_4_ severity, the size of AHI_4_ strata is controlled through enrollment limits, with each AHI_4_ stratum representing ∼20–40% of participants. During the initial week of the study, participants receive either a lower dose of AD109 (2.5/37.5 mg, referred to as the run-in dose) or a matched placebo. This phase is followed by the administration of the target dose (2.5/75 mg) for the duration of the study. Study medication is taken orally, once daily at bedtime.

These studies are conducted in accordance with the currently approved protocol, International Conference on Harmonization Good Clinical Practice (ICH GCP guidelines), and all applicable regulatory requirements. All participants provided written informed consent before participating in the trials.

#### 2.1.1 Study organization

Apnimed Inc. is the sponsor of the LunAIRo and SynAIRgy clinical trials. Apnimed transferred some of its obligations for the trials to a clinical research organization (CRO, Syneos Health, Morrisville, NC), responsible for several tasks including the construction and management of the study database and materials, ongoing onsite study site monitoring to ensure participant safety, data integrity, adherence to regulations, and generation of statistical reports for both the sponsor and the Data Monitoring Committee (DMC). The studies are conducted with the support of a PSG scoring center (Sleep Strategies, Ottawa, ON) responsible for centralized PSG scoring blinded to treatment assignment for both screening and outcome assessment and generation of standardized PSG variables. PSGs were manually scored by eight different technicians. Each participant's baseline and on-treatment PSGs were evaluated by the same scorer to maintain consistency. Regular calibration meetings were held to harmonize scoring practices and minimize inter-rater variability.

Clinical sites are each headed by an experienced investigator and are responsible for recruitment and follow-up of participants. A total of 64 sites have been identified in the U.S. for LunAIRo (Supplemental Table 1) and 73 sites have been identified in both the U.S. and Canada for SynAIRgy (Supplemental Table 2). Many sites were activated to meet the target enrollment. Additionally, substantial effort and outreach was conducted to ensure that a balanced ratio of male and female participants were included in the studies. Moreover, these studies aimed to recruit a participant population that was representative of the diverse demographic and ethnic composition of the U.S.

All sites are certified by Sleep Strategies for PSG capabilities. Sleep Strategies is also monitoring PSG data quality during the trial. PROs are gathered during clinic visits using an electronic application accessible via a tablet and provided by Clinical Ink (Winston-Salem, NC). Physician medical monitoring is provided by site investigators. An independent DMC, with expertise in sleep apnea, clinical trials, and biostatistics meets regularly to assess unblinded safety data and make recommendations for both trials. Measures to maintain quality control are outlined in the **Supplemental Methods**.

### 2.2 Study objectives

The primary objective of the LunAIRo and SynAIRgy trials is to demonstrate the efficacy of AD109 versus placebo for upper airway obstruction, as measured by the AHI_4_, among individuals across the full range of OSA severity, from mild to very severe, over a 26-week treatment period. Secondary objectives are to demonstrate efficacy of AD109 vs placebo on OSA symptoms including fatigue, one of the most common and bothersome OSA symptoms [[13], [14], [15]], and on measures of oxygenation which have been shown to correlate more strongly than AHI with adverse cardiovascular outcomes [16]. Additional objectives for the LunAIRo trial include exploring both long-term efficacy and safety endpoints over a 51-week placebo-controlled period, and exploring time points as early as 4 weeks to evaluate rapid onset of efficacy of AD109. Exploratory objectives for both trials involve in-depth examination of the effects of AD109 on sleep parameters, and additional measures of airway obstruction.

### 2.3 Participants

A summary of key inclusion and exclusion criteria is shown in Table 1. Enrollment targeted participants who are broadly representative of the adult OSA population. Potential participants for inclusion were comprised of both patients previously known to study sites and those responding to broad community outreach efforts specifically designed to reach a diverse population. Participants were enrolled in LunAIRo or SynAIRgy based on the closest location available to them. LunAIRo and SynAIRgy were performed in different centers, with the exception of a small number of sites that enrolled for SynAIRgy after the enrollment of LunAIRo was closed. No participant was enrolled in both studies.

**Table 1:** List of key inclusion and exclusion criteria for both LunAIRo and SynAIRgy trials.

Inclusion Criteria	Exclusion Criteria
**Age and Sex** • ≥18 years of age at the time of informed consent. **OSA History and Measures** • PSG criteria (collected at Visit 2) a. AHI_4_ (Hypopneas defined by 4% oxygen desaturation) of >5 b. ≤ 25% central or mixed apneas (as proportion of total apneas + hypopneas) c. PLM arousal index ≤15 • PROMIS-Fatigue Short Version 8a: raw score ≥17 at Visit 1^a^ • PAP failure or PAP refusal d. PAP failure is defined as no PAP use for ≥3 months prior to randomization, or return/removal of PAP device from the home. e. PAP refusal is defined as refusal of PAP after prior positive sleep study, or prior refusal of provider-recommended sleep study due to unwillingness to consider PAP. **Weight** • BMI between 18.5 and 40 kg/m^2^ for men, or 18.5 and 42 kg/m^2^ for women, inclusive.	**Medical Conditions** • Narcolepsy, restless legs syndrome requiring medication, REM sleep behavior disorder • Current bothersome symptoms of insomnia (difficulty initiating or maintaining sleep, as distinct from unrefreshing sleep or other symptoms attributable to OSA). Participants treated with insomnia can be enrolled if symptoms are controlled • Pierre Robin, Treacher Collins, or other craniofacial malformation syndrome, or grade ≥3 tonsillar hypertrophy. • Clinically significant or medically uncontrolled cardiovascular disease, or resting heart rate >100), untreated or unstable coronary artery disease, heart failure, cerebrovascular event or revascularization within 3 months. • Neuromuscular disorder, epilepsy, Parkinson, Alzheimer, or other neurodegenerative disease • Schizophrenia, schizoaffective disorder, generalized anxiety disorder or bipolar disorder according to DSM-5 criteria • Attempted suicide within 1 year prior to screening, or current suicidal ideation. • Severe or frequent constipation considered currently bothersome by the patient, or symptomatic gastric motility disorder. • Current bothersome symptoms of bladder outlet obstruction including difficulty initiating or maintaining urinary flow, straining, or sensation of incomplete bladder emptying • Active substance use disorder as defined in DSM-5, or other substance use that would present an unreasonable risk to the participant, or which would interfere with their participation in the study or confound study interpretation • Blood pressure >145/90 mmHg • A serious illness or infection • Clinically significant cognitive dysfunction • Narrow angle glaucoma • Women who are pregnant or nursing • History of chronic oxygen therapy, or implanted devices for the treatment of OSA • Use of mandibular advancement devices, nasal devices, or sleeping position devices during study

Abbreviations: AHI_4_, apnea-hypopnea index based on 4% hypopnea desaturation; BMI, body mass index; DSM-5, Diagnostic and Statistical Manual of Mental Disorders, 5th Edition; OSA, obstructive sleep apnea; PAP, positive airway pressure; PLM, periodic limb movement; PSG, polysomnography; REM, rapid eye movement.

a A PROMIS-Fatigue raw score of 17 is equivalent to a T-score of 50.4.

The targeted study population is adults (≥18 years old, no upper age limit) with OSA of any severity defined as an AHI_4_>5 events/h (hypopneas scored when associated with 4% desaturation) with ≤25% central or mixed apneas (as proportion of total apneas and hypopneas), and PLM arousal index ≤15 events/h on the screening PSG. Since the mechanism of action should apply, in theory, to all OSA patients, no limit to OSA severity is implemented initially, however caps could be implemented during the trial to ensure that the full range of OSA severities are well-represented in the trials. Only participants who refuse or are intolerant to PAP and with a raw PROMIS-Fatigue score ≥17 (fatigue level above average of the population; a raw score of 17 is equivalent to a T-score of 50.4) are eligible.

Potential participants with clinically significant or medically uncontrolled cardiovascular disease such as unstable atrial fibrillation, untreated or unstable coronary artery disease, heart failure, cerebrovascular events and resting heart rate >100 beats per min or blood pressure greater than 145/90 mmHg were excluded. Atomoxetine is metabolized by CYP2D6 and aroxybutynin by CYP3A4, and consequently, strong pharmacologic inhibitors of these metabolic enzymes are not allowed as concomitant therapy. Concomitant therapy with allowed and disallowed medications is listed in Table 2.

**Table 2:** Concomitant medications.

Allowed Medications	Disallowed Medications
• Benzodiazepines, Z-drugs, DORAs, and trazodone • Antihypertensives (except central agonist) • Statins, • Gabapentin, and pregabalin • SSRIs, PPI, histamine H2 receptor blockers, antacids, and proton pump inhibitors • Non-sedating antihistamines, • Melatonin, NSAIDs and acetaminophen • Anti-asthmatics, antibiotics, and topical antifungals • Laxatives, immunomodulators, and erectile dysfunction drugs • Inhaled corticosteroid, and antidiabetics, including those associated with weight loss • Ocular hypotensives • Hormonal therapy • Thyroid medications • Anticoagulants • Gout medications • OTC topicals • Osteoporosis drugs	• MAOIs, NRIs, and TCAs, • Strong CYP2D6 inhibitors, and strong CYP3A4 inhibitors • St John's Wort, barbiturates, buspirone, opioids • Muscle relaxants (in people ages >65 years), pressor agents, central agonist antihypertensives, and amphetamines • Drugs used exclusively for weight loss • Dopaminergic or antihistaminergic antiemetics • Modafinil, armodafinil, solriamfetol, and pitolisant • Esketamine • Antipsychotics, • Oral anticholinergics, and cholinesterase inhibitors • More than occasional use of sedating antihistamines

Medications that do not have substantial effects on the central nervous system, respiration, or muscle activity are typically allowed even if not listed the column labeled: allowed medications. On a case-by-case basis with approval of the sponsor, a medication that is typically disallowed can be used to treat an intercurrent illness or condition that arises during the course of the study. Abbreviations: DORA, dual orexin receptor antagonists; MAOI, monoamine oxidase inhibitor; NRI, norepinephrine reuptake inhibitor; NSAID, nonsteroidal anti-inflammatory drug; OTC, over the counter; SSRI, selective serotonin reuptake inhibitor; TCA, tricyclic antidepressants; Z-drugs, nonbenzodiazepines.

### 2.4 Randomization and blinding

Eligible participants are centrally randomized during visit 3 (V3) to one of two parallel treatment arms using interactive response technology (IRT) accessed via internet browser (Suvoda, Conshohocken, Pennsylvania). Randomization is stratified according to baseline AHI_4_ (<15, 15 to 30, >30) and 8-item PROMIS-Fatigue raw score (<25 vs ≥ 25; a raw score of 25 is equivalent to a T-score of 58.5). To ensure representative distribution of baseline AHI_4_ severity, the size of AHI_4_ strata will be controlled through enrollment limits, with AHI_4_ <15 representing ∼20–40% of participants, AHI_4_ 15–30 representing ∼20–40% of participants, and AHI_4_ >30 representing ∼20–40% of participants.

Each participant is assigned a unique randomization number linked to study arm assignment. All study participants, investigators, site staff, staff at the PSG scoring center, staff at the CRO and sponsor staff are blinded to treatment assignment throughout the studies. Unblinding of a participant's study treatment assignment is only allowed if determined by the investigator to be necessary to protect participant safety. Knowledge of the participant's treatment assignment is closely restricted only to those individuals who need to know to protect patient safety, such as members of the Data Monitoring Committee (DMC).

### 2.5 Study procedures

A screening visit (V1) determines study eligibility based on non-PSG criteria, including clinical laboratory testing results. Activities during V1 include obtaining informed consent, assessing demographics, medical history, physical examination, and various tests, including urine drugs of abuse, pregnancy, 12-lead ECG, and PROMIS-Fatigue.

Visit 2 (V2) involves screening/baseline PSG and is read by the central PSG reading center for individuals meeting all enrollment criteria assessed at V1. The PSG and Patient-Reported Outcomes (PROs) conducted during V2 serve as the baseline for respective efficacy outcomes. PROs, including Epworth Sleepiness Scale (ESS), PROMIS-Fatigue, PROMIS v1 - Sleep Impairment short form 8a (PROMISE-SI), and patient global impression of severity (PGI-S) are assessed in the evening, while digital symbol substitution test (DSST), psychomotor vigilance test (PVT), and Verbal Learning Task (VLT) are assessed the morning after the PSG. V2 PSG data are sent to the central PSG reading center (Sleep Strategies), which notifies sites of eligibility status. Only eligible individuals proceed to V3.

Eligible participants in the LunAIRo trial, after V2, will be encouraged to undergo optional 24 h ambulatory blood pressure monitoring (ABPM) before the randomization visit (V3). The ABPM procedure is repeated after 4 weeks of treatment with AD109 or placebo. Blood pressure and other vital signs are otherwise monitored during each visit at the clinical site in both LunAIRo and SynAIRgy.

At V3, qualifying participants are randomized, instructed on the investigational medicinal product (IMP) dosing, and receive the IMP. The IMP is dispensed as a blister package of 7 run-in dose tablets and a bottle containing 33 target dose tablets. Participants take the run-in dose nightly at home and return with the bottle and empty blister pack at V4 for IMP accountability. Contact with participants is maintained throughout the study, with follow-up phone calls performed at specific days for monitoring adverse events (AEs) and concomitant therapy.

In-laboratory PSGs are performed after 4 and 26 weeks for SynAIRgy, and after 13, 26, and 51 weeks for LunAIRo. In addition to PSG visits, participants return to the site at least every 3 months with remaining IMP and any empty bottles, undergo IMP accountability, AE assessment, vital signs, weight, ECG, administration of PRO instruments, and receipt of additional IMP supply. Follow-up contact calls to collect AEs in between in-person visits are scheduled at least every 1 month.

The end of study visit occurs approximately 2 weeks after the cessation of IMP. Activities include monitoring AEs including specific assessment of symptoms potentially related to IMP discontinuation, vital signs, weight, ECG, and assessment of various PROs.

### 2.6 Study interventions

The intervention period is 51 weeks for the LunAIRo study and 26 weeks for the SynAIRgy study. All participants must be PAP intolerant or have refused PAP to enroll. Participants are not prohibited from initiating PAP during the trial if deemed by the participant's primary care provider to be in the participant's best medical interest. Dosing of AD109 or placebo can continue if PAP is initiated. Dose reduction is not permitted, and dosing is discontinued if the participant experiences intolerance to the assigned study drug. Participants who discontinue dosing will be encouraged to continue all other study activities for the entire duration of the trial to support an intention-to-treat analysis.

### 2.7 Study endpoints

#### 2.7.1 Efficacy

A complete list of the primary, and secondary objectives and endpoints is shown in Table 3 with assessment details provided in **Supplemental Methods**. The primary efficacy endpoint for both trials is the change from baseline in AHI_4_ at Week 26. Key secondary endpoints for both trials include the change from baseline in oxygen desaturation index based on 3% desaturation (ODI_3_) at Week 26, the change from baseline in hypoxic burden based on 4% desaturation (HB_4_) at Week 26, and the proportion of participants with ≥50% reduction in AHI_4_ at Week 26. The sleep apnea-related HB_4_ is an objective measurement related to the oxygen saturation associated with OSA. It is calculated as the area of the desaturation below the pre-obstructive event baseline and expressed as %desaturation∗minutes/hours of sleep.

**Table 3:** Objectives and endpoints of the LunAIRo and SynAIRgy trials.

Objectives	Endpoints	
Primary Objective • Compare efficacy on airway obstruction of AD109 vs placebo in participants with mild to severe OSA	Primary Endpoint	
	LunAIRo • Change from baseline in AHI_4_ at Week 26	SynAIRgy • Change from baseline in AHI_4_ at Week 26
**Secondary Objectives** • Compare efficacy on OSA symptoms and oxygenation of AD109 vs placebo in participants with mild to severe OSA	**Key Secondary Endpoints**	
	**LunAIRo** Assessed at Week 26: • Change from baseline in ODI_3_ • Change from baseline in HB_4_ • Proportion of participants with ≥50% reduction in AHI4 • Change from baseline in PROMIS-Fatigue T-score	**SynAIRgy** Assessed at Week 26: • Change from baseline in ODI_3_ • Change from baseline in PROMIS-Fatigue T-score • Change from baseline in HB_4_ • Change from baseline in PROMIS-Sleep Impairment T-score • Proportion of participants with ≥50% reduction in AHI_4_
	**Secondary Endpoints**	
	**LunAIRo** Assessed at Week 26: • Change from baseline in PGI-S for Fatigue • PGI-C for Fatigue • Change from baseline in ESS • Change from baseline in PROMIS-Sleep Impairment T-score	**SynAIRgy** Assessed at Week 26: • Change from baseline in PGI-S for Fatigue • PGI-C for Fatigue • Change from baseline in ESS
**Safety Objectives** • Evaluate the safety and tolerability of treatment with AD109 in participants with mild to severe OSA	**Safety Assessments** • Physical exam, vital signs, clinical laboratory assessment, ECG • Spontaneous adverse events, including during the post-dosing period • DSST • PVT • Verbal learning task • M-CSSA • MADRS-S • Spielberger	

Abbreviations: AHI_3a_, apnea-hypopnea index based on 3% hypopnea desaturation plus arousals; AHI_4_, apnea-hypopnea index based on 4% hypopnea desaturation; DSST, Digit Symbol Substitution Test; ECG, electrocardiogram; ESS, Epworth sleepiness Scale; HB4, hypoxic burden based on 4% hypopnea desaturation; MADRS-S, self-reported Montgomery-Asberg depression rating scale; M-CSSA, Modified cocaine selective severity assessment; ODI_3_, oxygen desaturation index based on 3% hypopnea desaturation; OSA, Obstructive Sleep Apnea; PGI-C, patient global impression change; PGI-S, patient global impression severity; PROMIS, Patient Reported Outcomes Measurement Information System; PSG, polysomnography; PVT, psychomotor vigilance test; Spielberger, short version of Spielberger anxiety inventory.

An additional key secondary endpoint for both trials is the change from baseline in PROMIS-Fatigue T-score at Week 26. Such endpoints developed under the PROMIS initiative addressed a need for rigorously designed and validated PRO measurement tools that have utilized recent advances in information technology, psychometrics, and qualitative, cognitive, and health survey research. The PROMIS initiative developed new ways to measure PROs for several symptoms such as fatigue, sleep quality and daytime functioning that have a major impact on quality-of-life across a variety of chronic diseases, including OSA [17]. Fatigue is one of the most common patient-reported symptoms in relationship to sleep apnea and disturbed sleep, together with sleepiness [18]. The change from baseline in PROMIS-Sleep Impairment T-score at Week 26 is also a key secondary endpoint in SynAIRgy.

Other secondary endpoints in both trials include the change from baseline in the PGI-S score for fatigue at Week 26, the patient global impression of change (PGI-C) score for fatigue at Week 26, and the change from baseline in ESS at Week 26. The change from baseline in PROMIS-Sleep Impairment T-score at Week 26 is an additional secondary endpoint in LunAIRo.

#### 2.7.2 Safety

The safety monitoring for the two clinical trials prioritizes the established safety profiles of its components, aroxybutynin and atomoxetine, alongside data from earlier phase trials. Safety assessments encompass physical exams, vital signs, and the documentation of AEs, serious adverse events (SAEs), and any discontinuations. Adverse events of special interest include urinary issues, cardiovascular symptoms, suicidal ideation, daytime sleepiness, motor vehicle accidents, and anticholinergic effects, with a focus on recording patient-reported experiences. Participants that discontinue IMP are encouraged to continue all study assessments until the study's completion.

A Data Monitoring Committee (DMC), comprised of individuals with relevant expertise with no direct relationship to the study, are appointed by Apnimed. The DMC may consider recommending early termination of studies on safety grounds.

### 2.8 Statistical power

The sample size of each study (LunAIRo and SynAIRgy) has been determined based on achieving approximately 90% power within each study for both the primary endpoint (the change from baseline in AHI_4_ at Week 26) and the key secondary endpoint (change from baseline in PROMIS-Fatigue T-score at Week 26), under a 2-sided 5% significance level, after adjustment for multiplicity.

In a previous Phase 2 study (MARIPOSA), the standard deviation of the placebo group change from baseline in AHI_4_ was approximately 9.0. Based on the observed dropout rates in MARIPOSA, it is assumed that the dropout rate in the placebo group will be 5% and the dropout rate in the AD109 group will be 25%. For the primary analysis, missing data will be handled under a missing-not-at-random assumption by using multiple imputation from the control group. Based on the assumed standard deviation, anticipated dropout rates and planned method of handling missing data, the required sample size was estimated using simulation. A total of 640 participants (320/group) will provide >98% power to detect a treatment difference of ≥ 4 points between AD109 and placebo for the change in AHI_4_.

In the Phase 2 MARIPOSA study, the placebo group's standard deviation in PROMIS-Fatigue T-score change from baseline was 8.6. Anticipated dropout rates (5% for placebo, 25% for AD109) and a missing-not-at-random assumption for missing data using multiple imputation from the control group were considered. With these assumptions, simulation demonstrated that 640 participants (320/group) would provide approximately 90% power to detect a treatment difference of 2.5 points and approximately 95% power to detect a treatment difference of 2.75 points.

### 2.9 Statistical analysis plan

Efficacy will be evaluated in the intention-to-treat (ITT) and modified intention-to-treat (MITT) sets. The ITT includes all participants who are randomized and receive at least 1 dose of study medication. The MITT includes all participants who are randomized and receive at least 1 dose of study medication and have one post baseline PSG assessment on treatment. The primary analysis methodology will utilize a restricted maximum likelihood (REML) based mixed model for repeated measurements (MMRM) in combination with the Newton Raphson Algorithm. Analyses will include treatment, AHI_4_ strata, time and the treatment by time interaction as a fixed effects and baseline score as a covariate. An unstructured covariance matrix will be used to model the within participant error. If the model fails to converge with an unstructured covariance matrix, a compound symmetric covariance matrix will be used. In the case that a structured variance-covariance matrix is used to enable the model to converge, the "sandwich" estimator of the variance-covariance matrix will be employed. The Kenward-Roger approximation will be used to estimate the denominator degrees of freedom. The analysis will be implemented using the MIXED procedure in SAS. The least squares (LS) means, and treatment difference in LS means for Week 26 will be reported along with the corresponding 95% confidence intervals and the p-values for the treatment group comparisons. The primary comparison will be the contrast between treatment groups at Week 26. All data collected, regardless of adherence to investigational intervention, and initiation of PAP will be used in the analysis.

Analysis of continuous secondary endpoints will be analyzed using the same methods as outlined for the primary endpoint. The proportion of participants with ≥50% reduction in AHI_4_ at Week 26 will be compared between treatment groups using the stratified Cochran-Mantel Haenszel procedure, controlling for randomization strata. An estimate of the unadjusted response rates in AD109 and placebo, the adjusted risk difference of the response rates between AD109 vs placebo, along with the 2- sided 95% CI for the risk difference will be presented. The Mantel-Haenszel estimate of the adjusted risk difference, and confidence limits for the adjusted risk difference will be based on the Mantel-Haenszel stratum weights [19] and the Sato variance estimator [20].

As there is a single primary endpoint, with a single primary hypothesis test, no adjustment for multiplicity is needed for the primary hypothesis test, which will use a two-sided 5% significance level. Adjustment for multiplicity of the key secondary endpoints will be conducted through a closed testing approach, where each key secondary endpoint will be tested in a pre-specified order at Week 26 as follows for LunAIRo: 1) Change from baseline in ODI_3_, 2) Change from baseline in HB4, 3) Proportion of participants with ≥50% reduction in AHI_4_ 4) Change from baseline in PROMIS-Fatigue T-score, and for SynAIRgy: 1) Change from baseline in ODI_3_, 2) Change from baseline in PROMIS-Fatigue T-score, 3) Change from baseline in HB_4_, 4) Change from baseline in PROMIS-Sleep Impairment T-score, 5) Proportion of participants with ≥50% reduction in AHI_4_. Secondary endpoints will be tested using the hierarchical structure using a two-sided 5% significance level until an endpoint is found to be not significant, or all secondary endpoints have been tested. No adjustment for multiplicity will be made for non-key secondary endpoints.

## 3 Demographics and baseline disease characteristics

The demographic and baseline disease characteristics of the two trials are presented in Table 4. A total of 660 and 646 participants were randomized to AD109 or placebo in LunAIRo and SynAIRgy, respectively. At baseline, mean (SD) AHI_4_ was 24 (18) events/hr in LunAIRo and 22 (11) events/hr in SynAIRgy. Randomized participants exhibit baseline AHI_4_ scores across the broad spectrum of OSA disease severity. The racial and ethnic diversity along with balanced ratio of female-to-male participants is representative of the general population of people living with OSA. Similarly, the symptom profiles reflect a typical sleep clinic referral population.

**Table 4:** Demographics and baseline disease characteristics of LunAIRo and SynAIRgy phase 3 trials.

Characteristic	LunAIRo N = 660	SynAIRgy N = 646
Age (yrs), mean (SD)	57 (10)	57 (11)
BMI (kg/m^2^), mean (SD)	32.1 (4.8)	32.3 (5.0)
Sex, n (%)		
Female	303 (46)	317 (49)
Male	357 (54)	329 (51)
Race, n (%)		
American Indian or Alaskan Native	13 (2.0)	7 (1.1)
Asian	23 (3.5)	48 (7.4)
Black or African American	113 (17.1)	132 (20.4)
Native Hawaiian or Other Pacific Islander	7 (1.1)	4 (0.6)
Multiracial or Other	8 (1.2)	5 (0.8)
White	492 (74.5)	434 (67.2)
Not Reported/Unknown	4 (0.6)	16 (2.5)
Ethnicity, n (%)		
Hispanic or Latino	111 (16.8)	136 (21.1)
Not Hispanic or Latino	539 (81.7)	495 (76.6)
Not Reported/Unknown	10 (1.5)	15 (2.3)
AHI_4_, mean (SD)	24 (18)	22 (11)
AHI_4_ severity, n (%)		
Mild, AHI_4_ 5–<15	248 (37.6)	222 (34.4)
Moderate, AHI_4_ 15–<30	216 (32.7)	274 (42.4)
Severe, AHI_4_ ≥30	196 (29.7)	150 (23.2)
ODI_3_, mean (SD)	32 (19)	29 (13)
HB4, mean (SD)	55 (68)	43 (40)
PROMIS-Fatigue T-score, mean (SD)	59 (7)	59 (7)
PROMIS-SI T-score, mean (SD)	59 (7)	59 (7)
ESS, mean (SD)	10.2 (4.8)	10.1 (4.7)

Abbreviations: AHI_4_, apnea-hypopnea index with 4% desaturation criterion; BMI, body mass index; ESS, Epworth Sleepiness Scale; HB4, hypoxic burden with 4% desaturation criterion; PGI-S, Patient Global Impression of Severity; PROMIS-Fatigue, Patient Reported Outcomes Measurement Information System – Fatigue; PROMIS-SI, Patient Reported Outcomes Measurement Information System – Sleep Impairment; SD, standard deviation.

## 4 Discussion

The LunAIRo and SynAIRgy trials represent the most extensive effort to date to evaluate the efficacy and safety of an oral medication taken at bedtime aimed at addressing the primary cause of OSA—neuromuscular dysfunction resulting in obstruction of the upper airway due to sleep-related decreased upper airway muscle tone. The combination of atomoxetine and aroxybutynin is designed to directly address the neuromuscular pathophysiology of OSA and has demonstrated significant reductions in AHI across multiple clinical studies of up to 1 month in duration, including the recent Phase 2 MARIPOSA study, a placebo-controlled dose-finding trial that informed the design of the current phase 3 trials [12,21].

The primary efficacy endpoint in most OSA trials is the AHI which historically has been the principal biomarker for the disease [[22], [23], [24], [25]]. The consequences of sleep disruption and hypoxemia in OSA are diverse, such that different metrics can reflect different impacts on patients’ well-being and functionality. Importantly, chronic intermittent hypoxia has been linked to an increased risk of OSA-related comorbidities. Alongside AHI, we are also measuring changes in ODI_3_ and HB_4_ as key secondary efficacy endpoints in both LunAIRo and SynAIRgy. HB_4_ recently has been introduced as an alternative objective metric to assess OSA severity. Compared to AHI_4_, that only considers the frequency of obstructive events (events/hour of sleep), the HB_4_ also incorporates the length and the depth of the apnea/hypopnea-related chronic intermittent hypoxia during sleep [16]. This metric has demonstrated a stronger correlation with cardiovascular mortality than AHI in recent epidemiological cohort analyses [16].

The trials each incorporate PROMIS-Fatigue, PROMIS Sleep Impairment, and ESS. Fatigue is one of the most common and most bothersome symptoms of OSA and is a distinct symptom from sleepiness [13]. A systematic evaluation of key elements that affect quality of life in individuals with OSA identified fatigue or decreased energy in 71% and 80%, respectively [14]. The complaint of “falling asleep if not stimulated or active” was reported less frequently, at 61%. Excessive daytime sleepiness (EDS) is commonly associated with OSA [26,27] and has been linked to higher comorbidity, impairment in productivity [28] and increased risk of motor vehicle accidents in OSA [29]. However, excessive daytime sleepiness (ESS>10) may occur in half or fewer of those with OSA, with typical reported prevalence between 15% and 50% [30]. The Epworth Sleepiness Scale (ESS) is commonly used to evaluate EDS in OSA, although about half of even severe OSA patients score within the normal range measures [27]. Fatigue has been reported to improve significantly among patients without evidence of baseline sleepiness, and among patients with only mild OSA [15]. PROMIS-Fatigue may provide increased sensitivity to symptom improvement in OSA [31], particularly in the substantial proportion of OSA patients without EDS [32].

The trial population consists of participants who have refused or are intolerant to PAP treatment. Given the study's duration, participants are permitted to initiate PAP during the trial if considered by the participant and their primary care provider to be in the participant's best interest. To minimize dropouts and confounding factors, carefully selected participants with little inclination to use PAP will be enrolled.

In conclusion, the LunAIRo and SynAIRgy phase 3 trials will be pivotal in advancing our understanding of the treatment of OSA with AD109. These trials are unique in their comprehensive approach, assessing both objective and subjective outcomes over an extended period of 51 weeks and 26 weeks, respectively. The findings from these trials are anticipated to significantly impact the management of OSA, particularly for the large number of patients who are unable or unwilling to use PAP therapy and may potentially represent a new standard of care for this common and serious sleep-related breathing disease.

## CRediT authorship contribution statement

**Luigi Taranto-Montemurro:** Writing – review & editing, Writing – original draft, Supervision, Project administration, Methodology, Conceptualization. **Sanjay R. Patel:** Writing – review & editing, Investigation, Conceptualization. **Patrick J. Strollo:** Writing – review & editing, Investigation, Conceptualization. **John Cronin:** Writing – review & editing, Methodology, Conceptualization. **John Yee:** Writing – review & editing, Methodology, Conceptualization. **Huy Pho:** Writing – review & editing, Formal analysis. **Andrea Werner:** Writing – review & editing, Supervision. **Ron Farkas:** Writing – review & editing, Methodology, Data curation, Conceptualization.

## Funding statement

These studies were supported by Apnimed, Inc.

## Declaration of competing interest

LT-M, JC, JY, HP, AW, RF are Apnimed employees.

SRP has served as a consultant for Apnimed, Bayer Pharmaceuticals, Philips Respironics, and SleepRes and has received grant support through his institution from Bayer Pharmaceuticals and Philips Respironics.

PJS has served as a consultant for Apnimed, Inspire Medical Systems, Cryosa, Somnomed, Philips Respironics, WisperSom, Biologix, Emmi Solutions, Zoll Medical Corporation, Restora, XII Medical and has received grant support through his institution from ResMed, Inspire Medical Systems, and Zoll Medical. He is a Non-Executive Director of Belluscura

## Data Availability

The data that has been used is confidential.
