# Supplementary material for: Aroxybutynin and atomoxetine (AD109) for the treatment of obstructive sleep apnea: Rationale, design and baseline characteristics of the phase 3 clinical trials
Source: Contemp Clin Trials Commun. 2025 Aug 17;47:101538. doi: 10.1016/j.conctc.2025.101538 (PMC12490522; doi:10.1016/j.conctc.2025.101538)
Supplement: Multimedia component 1 [file mmc1.docx]

**Supplemental Information**

**Aroxybutynin and Atomoxetine (AD109) for the Treatment of Obstructive Sleep Apnea: Rationale, Design and Baseline Sample Characteristics of the Phase 3 Clinical Trials**

Luigi Taranto-Montemurro^1^, Sanjay R. Patel^2^, Patrick J. Strollo, Jr.^2^, John Cronin^1^, John Yee^1^, Huy Pho^1^, Andrea Werner^1^ and Ron Farkas^1^.

1. Apnimed, Inc., Cambridge, MA, USA
2. University of Pittsburgh, PA, USA

**Supplemental methods**

**Open-label Extension**

The open-label extension (OLE) functions as a continuum for participants concluding both clinical trials. The primary objectives of this extension study are twofold: first, to provide eligible participants with continued access to AD109, and second, to systematically gather long-term safety data. Eligibility is open to all participants, regardless of whether they still meet the inclusion criteria of the parent studies, except in cases where participation may pose a safety risk based on the individual's current medical condition or ongoing therapies, as determined by the investigators. This extension study plays a pivotal role in ensuring the comprehensive evaluation of AD109's safety profile over an extended 1-year period, and advances our understanding of its tolerability as a long-term treatment for OSA*.*

**Quality Control**

Quality Control in these studies involves multiple levels of training, monitoring, and feedback activities. These include centrally trained site coordinators, sleep technicians, investigators, sleep scorers and study monitors. Study procedures require site training or certification, and staff must be trained before interacting with study participants. Specific requirements vary by procedure but generally entail documenting successful performance during central training and submission of tests such as PSG recordings meeting quality and completeness standards as part of site qualification.

After initial certification, ongoing monitoring of each site's performance takes place, including of PSG quality, with regular feedback provided to sites to address quality issues and to enhance consistency in performance.

All participant data related to the study is recorded on electronic case report forms (eCRFs) unless electronically transmitted to the CRO (e.g., PSG data, laboratory tests, psychometric tests). Study monitors continuously verify source data to ensure accuracy, completeness, and verifiability from source documents. They also ensure the safety and rights of participants are protected and that the study is conducted in accordance with the currently approved protocol, ICH GCP, and all applicable regulatory requirements.

**Patient reported outcome measures and psychometrics tests**

The Epworth Sleepiness Scale (ESS) is a self-administered questionnaire with 8 questions. Participants are asked to rate, on a 4-point scale (0-3), their usual chances of dozing off or falling asleep while engaged in 8 different activities in recent times. The ESS score (the sum of 8 item scores, 0-3) can range from 0 to 24. The higher the ESS score, the higher that person’s average sleep propensity in daily life, or their ‘daytime sleepiness’. The questionnaire takes approximately 2 or 3 minutes to answer.

PROMIS (Patient-Reported Outcomes Measurement Information System) sleep impairment and fatigue measures were developed with modern psychometric techniques including item response theory to assess various self-reported aspects of sleep and daytime impairment. Items are based on 5-point scales, either frequency or intensity, with higher scores corresponded to more severe or worse symptoms. The short versions for PROMIS Fatigue and Sleep impairment 8a will be used in these trials.

Patient Global Impression of Change (PGI-C)/Patient Global Impression of Severity (PGI-S) for Fatigue are each single-item scales of the patient’s global, i.e., overall impression of either change from pre-treatment in fatigue (PGI-C) or severity of fatigue proximate to the timepoint measured (PGI-S).

The Digit Symbol Substitution Test (DSST) measures a range of cognitive and motor operations including motor speed, attention, visuo-perceptual functions, and some aspects of associative learning. The DSST requires participants to match symbols to numbers according to a key located on the tablet computer screen. The DSST is used in this study primarily as a safety measure for psychomotor impairment related to residual next-day effects of study drug dosed at bedtime.

The Psychomotor Vigilance Test (PVT) measures sustained attention and reaction time over 10 minutes. Participants are instructed to press the screen of a tablet computer as quickly as possible after a visual cue appears. In addition to reaction time, the test records lapses, in which the screen is not pressed in response to a cue, and false-positives in which the screen is pressed prior to appearance of the cue.

The Verbal Learning Task (VLT) measures the ability to store words in working memory and long-term memory. In the learning phase, participants are instructed to pay attention to a list of one and two syllable words that are presented sequentially on a display, and to verbally repeat as many words as they can remember from the list. This cycle of presenting and repeating the same words is repeated three times. The recall phase is done after a prescribed delay. Participants are asked to verbally recall as many of the words as they can remember from the original list of words presented during the learning phase.

The DSST, PVT and VLT are performed on a tablet using BrainBaseline^TM^ technology.

**Twenty-four hours ambulatory blood pressure monitoring**

Lunairo participants will be asked to perform an optional 24-ambulatory blood pressure monitoring (ABPM) at baseline and after 30 days of treatment. Participants performing 24hr ABPM (Spacelabs) come to the site ~24hr prior to Visit 3 and after 30 days, before Visit 4 to be fitted with the ABPM device. Participants resume normal daily activities for the remainder of the day and overnight and return to the site the next day for device removal by site staff followed by the remainder of Visits 3 and 4 activities. Blood pressure is assessed every 30 minutes during the monitoring time. The report from the ABPM provides daily average of systolic, diastolic and mean blood pressure, as well as daytime (8:00-20:00) and nighttime (20:00-8:00) values.

**Supplemental Tables**

**Table S1.** List of LunAIRo Study Sites

| **Principal Investigator** | **Clinical Trial Site** | **Location** |
| --- | --- | --- |
| James Andry | Sleep Therapy Research Center | San Antonio, TX |
| Francis Averill | St. Francis Medical Institute | Clearwater, FL |
| Anup Banerjee | Clinical Research of Gastonia | Gastonia, NC |
| Mira Baron | Palm Beach Research ClinEdge-PPDS | West Palm Beach, FL |
| David Billmeier | Arrow Clinical Trials | Daytona Beach, FL |
| Bruce Corser | Sleep Management Institute | Cincinnati, OH |
| Luis De La Cruz | Velocity Clinical Research - Greenville | Greenville, SC |
| Robert Doekel | Sleep Disorders Center of Alabama | Birmingham, AL |
| Christopher Drake | Henry Ford Health | Novi, MI |
| Ryan Drake | NeuroScience Research Center, LLC | Canton, OH |
| Faisal Fakih | Clinical Site Partners, LLC dba Flourish Research | Winter Park. FL |
| Daniel Garber | Research Carolina Elite | Denver, NC |
| Mark Goetting | Western Michigan University Homer Stryker M.D. School of Medicine | Kalamazoo, MI |
| Silvana Gonzalez Reiley | Ivetmar Medical Group LLC | Miami, FL |
| Harly Greenberg | Northwell Health Physician Partners | New Hyde Park, NY |
| John Harsh | Alpine Clinical Research Center | Boulder, CO |
| John Hudson | FutureSearch Trials of Neurology | Austin, TX |
| Monika Jaffe | Chicago Research Center Inc | Chicago, IL |
| Kimball Johnson | CenExel iResearch | Decatur, GA |
| Karin Johnson | Baystate Medical Center, Baystate Sleep Medicine | Springfield, MA |
| Thomas Kaelin | Lowcountry Lung and Critical Care | Charleston, SC |
| Raj Karunakara | Renstar Medical Research | Ocala, FL |
| Venkatesh Krishnamurthy | UPMC Comprehensive Lung Center - Sleep Medicine | Pittsburgh, PA |
| Dennis Lacey | NeuroTrials Research | Atlanta, GA |
| Daniel Lorch | Teradan Clinical Trials LLC | Brandon, FL |
| Maria Mascolo | Centricity Research Rincon Pulmonology | Rincon, GA |
| David Maybee | Coastal Carolina Health Care | New Bern, NC |
| James Maynard | CTI Clinical Research Center | Cincinnati, OH |
| Lora McGill | Clinical Neuroscience Solutions | Memphis, TN |
| Michael McLeland | St Lukes Hospital | Chesterfield, MO |
| Jose Gabriel Medina-Smester | Medster Research, LLC | Valdosta, GA |
| Asefa Mekonnen | Velocity Clinical Research | Rockville, MD |
| Curtis Mello | Infinity Medical Research | N Dartmouth, MA |
| Edward Mezerhane | PharmaDev Clinical Research Institute, LLC | Miami, FL |
| Daniel Norman | Santa Monica Clinical Trials | Los Angeles, CA |
| Joseph Ojile | Clayton Sleep Institute | St. Louis, MO |
| Yogesh Paliwal | Empire Clinical Research | Pomona, CA |
| Andrew Pastewski | Nouvelle Clinical Research | Cutler Bay, FL |
| Terry Peery | Central Texas Neurology Consultants, PA | Round Rock, TX |
| A. Thomas Perkins | Meridian Clinical Research | Raleigh, NC |
| James Perlstrom | Site 1: Sleep Disorders Centers of the Mid-Atlantic | Glen Burnie, MD |
|  | Site 2: Sleep Disorders Centers of the Mid-Atlantic | Vienna, VA |
| Dawid R. Rechul | Maimonides Sleep Arts and Sciences | Albuquerque, NM |
| Larry Reed | Healthcare Research Network | Hazelwood, MO |
| Steven Reynolds | CenExel CNS | Los Alamitos, CA |
| Asim Roy | Ohio Sleep Medicine Institute | Dublin, OH |
| Muhammad Salim | Chandler Clinical Trials | Chandler, AZ |
| Bart Sangal | Clinical Neurophysiology Services | Sterling Heights, MI |
| Vikas Sayal | Henderson Clinical Trials | Henderson, NV |
| Andrew Schreiber | SDS Clinical Trials Inc. | Santa Ana, CA |
| Subramaniam Seetharaman | Northwest Research Center | Portland, OR |
| Craig Shapiro | Cenexel Research Centers of America - Hollywood | Hollywood, FL |
| Magdy Shenouda | Clinilabs, Inc | Eatontown, NJ |
| Steve Sitar | Orange County Research Institute | Anaheim, CA |
| Jefrey Start | Great Plains Health | North Platte, NE |
| Thomas Stern | Advanced Respiratory and Sleep Medicine, LLC | Huntersville, NC |
| Stephen Thein | Pacific Research Network | San Diego, CA |
| Robert Thomas | Neurocare, Inc | Newton, MA |
| Ian Weir | Nuvance Health | Norwalk, CT |
| David Weiss | Mountain View Clinical Research, Inc. | Denver, CO |
| Charles Wells, Jr. | Sleep Practitioners, LLC | Macon, GA |
| Paul Wylie | Preferred Research Partners, Inc. | Little Rock, AR |

**Table S2.** List of SynAIRgy Study Sites

| **Principal Investigator** | **Clinical Trial Site** | **Location** |
| --- | --- | --- |
| Akinyemi Ajayi | Florida Pediatric Research Institute | Winter Park, FL |
| Bernadette Alejandrino | Providere Research Inc | West Covina, CA |
| Jerome Alonso | Canadian Sleep Consultants | Calgary, AB |
| Najib Ayas | Vancouver Coastal Health Research Institute | Vancouver, BC |
| Francisco Badar | Core Healthcare Group | Cerritos, CA |
| Jacob Coleman | Javara Inc - Tryon Medical Partners PLCC | Charlotte, NC |
| William Cooper | Javarra Inc-Privia Medical Group | Thomasville, GA |
| Bruce Corser | Sleep Management Institute (Intrepid Research) | Cincinnati, OH |
| Ronald Cridland | Medical Arts Health Research | Kelowna, BC |
| Dominick D'Aunno | The Heights Hospital (HDHeights) | Houston, TX |
| Matthew Davis | Neurology Specialists of Monmouth County | West Long Branch, NJ |
| Bertrand De Silva | Probate Clinical Research Corporation | Riverside, CA |
| Michael Downing | FutureSearch Trials of Dallas LP | Dallas, TX |
| Alaa El-Gendy | Florida Lung and Sleep Associates | Lehigh Acres, FL |
| Tomas Fiel | Fiel Family Sports Medicine - PC CCT Research | Tempe, AZ |
| Steven Geller | Centennial Medical Group | Elkridge, MD |
| James Geyer | Alabama Neurology & Sleep Medicine | Tuscaloosa, AL |
| Andrew Gould | Advanced ENT and Allergy | Louisville, KY |
| Nella Green | Exalt Clinical Research | Chula Vista, CA |
| Mario Guillen | Canvas Clinical Research | Lake Worth, FL |
| Hermandeep Singh | Sleep Medicine Specialists of California (TriValley Sleep Center) | San Ramon, CA |
| John Hemmersmeier | South Ogden Family Medicine CCT Research | South Ogden, UT |
| John Hudson | FutureSearch Trials of Neurology | Austin, TX |
| Monica Jaffe | Chicago Research Center | Chicago, IL |
| Thomas Jarrett | Peters Medical Research, LLC | High Point, NC |
| John Khoury | Abington Neurological Associates | Abington, PA |
| John Kimoff | McGill University Health Centre | Montreal, QC |
| Oleg Kouskov | St. Lukes Clinic - Idaho Pulmonary Associates | Meridian, ID |
| Michael Lacey | NeuroTrials Research | Atlanta, GA |
| Judith Leech | West Ottawa Sleep Centre | Ottawa, ON |
| David Lesch | Georgia Neurology and Sleep Medicine Associates | Suwanee, GA |
| Michael Lillestol | Lillestol Research LLC | Fargo, ND |
| Reinero Linares - Mera | JSV Clinical Research Study Inc | Tampa, FL |
| Alan Lowe | AMNDX | Markham, ON |
| Kinjal Madhav | Profound Research LLC (North County Neurology Associates) | Carlsbad, CA |
| David Marks | Element Research Group | San Antonio, TX |
| Ronald Mayfield | Tribe Clinical Research LLC | Greenville, SC |
| James Maynard | CTI CRC | Cincinnati, OH |
| Jessica McCoun | Atlanta Center for Medical Research LLC | Atlanta, GA |
| Tatyana Miroshnikova | Clinical Neuroscience Solutions, Inc - Orlando | Orlando, FL |
| Rizwana Mohseni | Catalina Research Institute | Montclair, CA |
| Andrew Pastewski | Nouvelle Clinical Research | Cutler Bay, FL |
| Paresh Patel | Lone Star Lung and Sleep Clinic | Houston, TX |
| Susheel Patil | University Hospital of Cleveland Medical Center | Cleveland, OH |
| Nirupa Paulraj | Las Vegas Clinical Trials | North Las Vegas, NV |
| Enrique Pelayo | Advanced Medical Research Institute | Miami, FL |
| Dena Petersen | Noble Clinical Research | Tucson, AZ |
| Alec Platt | Respiratory Specialists | Wyomissing, PA |
| Lew Pliamm | Canadian Phase Onward Inc. | Toronto, ON |
| Bruce Rankin | Accel Research Sites | Deland, FL |
| Syed Raza | Revive Research Institute | Lathrup Village, MI |
| Anne Romaker | UC Health Sleep Medicine | Cincinnati, OH |
| Mark Rosenthal | Clinical Site Partners | Miami, FL |
| Eugene Ryan | Chattanooga Research & Medicine PLLC | Chattanooga, TN |
| Hector Sanchez | PMG Research of Wilmington | Wilmington, NC |
| Andrew Schreiber | SDS Clinical Trials | Santa Ana, CA |
| Sonja Schuetz | University of Michigan | Ann Arbor, MI |
| Sudhir Sehgal | Huntsville Research Institute LLC | Huntsville, TX |
| Colin Shapiro | Sleep and Alertness Center | Toronto, ON |
| Craig Shapiro | Cenexel RCA - Hollywood | Hollywood, FL |
| Gerald Shockey | Desert Clinical Research - CCT Research | Mesa, AZ |
| Sushil Singhi | OnSite Clinical Solutions LLC/Carolina Cardiology Associates P.A. | Rock Hill, SC |
| Steve Sitar | Orange County Research Institute | Anaheim, CA |
| Eileen Sloan | Toronto Sleep Institute | Toronto, ON |
| Damien Stevens | The University of Kansas Medical Center | Kansas City, KS |
| Kenneth Stiel | Foothills Research Center - CCT Research | Phoenix, AZ |
| Masayoshi Takashima | Houston Methodist/Weill Cornell Medical College | Houston, TX |
| Stephen Thein | Pacific Research Network | San Diego, CA |
| Patrick Whitten | OSF HealthCare Saint Francis Medical Center | Peoria, IL |
| Paul Wylie | Preferred Research Partners | Little Rock, AK |
| Dragos Zanchi | Meris Clinical Research | Brandon, FL |

**Table S3.** List of LunAIRo and SynAIRgy Vendors

| **Clinical Research Organization (CRO)**  Syneos Health, LLC  Morrisville, NC |
| --- |
| **PSG Scoring**  Sleep Strategies  Ottawa, ON, Canada |
| **ePRO**  Clinical Ink  Winston Salem, NC |
| **Clinical Safety Laboratory**  PPD Global Central Labs, LLC  Chicago, Illinois |
| **Electronic Data Capture (EDC)**  Medidata Solutions, Inc:  New York, NY |
| **Cardiac Safety**  Clario  Philadelphia, PA |
| **IRT Investigational Product Management**  Suvoda  Conshohocken, PA |
| **Patient Payment Solutions**  Greenphire  King of Prussia PA |
| **Subject Recruitment Services**  Galen Patient Recruitment  East Greenwich, RI  SubjectWell  Austin, TX |
